# Incidence of genetic polymorphisms involved in lipid metabolism among Chinese patients with osteonecrosis of the femoral head

**DOI:** 10.3109/17453670903025378

**Published:** 2009-06-01

**Authors:** Wei He, Keda Li

**Affiliations:** Department of Orthopaedics, First Affiliated Hospital of Guangzhou, University of Traditional Chinese Medicine, BaiYun DistrictGuangzhouChina

## Abstract

**Background and purpose** Corticosteroid treatment is associated with osteonecrosis of the femoral head (ON) in certain patients. The degree of drug sensitivity in general is governed by genetic variation between individuals. We investigated the relationship between ON and the presence of different alleles of the cytochrome P450 gene *(CYP3A4)*, the product of which metabolizes corticosteroids, and of the P-glycoprotein (P-gp) gene *(ABCB1),* the product of which modulates cellular uptake of corticosteroids, to determine whether patients with certain alleles may be at higher risk of ON after corticosteroid treatment.

**Methods** We studied 31 patients from Guangdong, China who were both treated with corticosteroid therapy and developed ON, and 17 corticosteroid-therapy patients without ON. Patient DNA was screened for known polymorphisms in the *CYP3A4* gene (CYP3A4*4, CYP3A4*5, CYP3A4*6) and the P-gp gene *ABCB1* (mutations C3435T, G2677T/A).

**Results** The majority (20/31) of the corticosteroid-treated patients who developed ON were heterozygous for *ABCB1,* whereas only 3/17 without ON were heterozygous. Statistical significance was observed between the ON and the control groups for the *ABCB1* G2677T/A polymorphism. Analysis of haplotypic frequencies indicated significant linkage disequilibrium between the two *ABCB1* polymorphisms, C3435T and G2677T/A (D' = 0.034). No *CYP3A4* polymorphisms were detected in any of the patients.

**Interpretation** Patients carrying an *ABCB1* polymorphism had a higher risk of having corticosteroid-associated ON than those with wild-type genotypes. This statistically significant association conflicts with previous studies, possibly due to different sampling methods. Knowing which genetic backgrounds are most strongly associated with corticosteroid-associated ON provides a method of screening for patients who are most at risk of developing ON.

## Introduction

Non-traumatic osteonecrosis (ON) of the femoral head is the gradual destruction of the femur head due to loss of blood flow and osteocyte death; it is characterized by disruption of intravascular blood flow, cellular toxicity, and impaired differentiation of mesenchymal cells, ultimately leading to bone death (Lieberman et al. 2003). Both arterial circulation—compromised by femoral neck fracture or arterial thrombosis/apoptosis from systemic diseases (e.g. lupus erythematous and hemoglobinopathies) (Mankin 1992)—and high intraosseous pressure, resulting from prolonged corticosteroid administration or ethanol abuse, obstruct blood flow in the femoral head and increase marrow cellularity and fat (Drescher et al. 2006). Although discreet pathogenetic mechanisms have yet to be identified in ON, corticosteroid and ethanol abuse have a profound effect on physiological bone turnover and oxygenation, and are associated with more than 90% of all osteonecrosis cases (Schroer 1994). The varying frequency and the clinical course of ON development in patients taking large doses of corticosteroids have been increasingly attributed (in part) to the presence of single nucleotide polymorphisms (SNPs) in the DNA sequences of genes encoding metabolic enzymes such as P-glycoprotein (P-gp) and cytochrome P450 (Asano et al. 2003b).

P-gp is an ATP-dependent membrane efflux pump that maintains intracellular drug and xenobiotic concentrations below cytotoxic levels (Tsuji et al. 1997). P-gp is encoded by the multidrug-resistance 1 *(MDR1)* gene, also known as the ABC transporter B1 *(ABCB1)* gene (Sakaeda et al. 2002). Corticosteroids are well established to be a P-gp substrate (Saeki et al. 1993). Numerous SNPs in the *ABCB1* gene have been identified, two of which (C3435T and G2677T/A) have been widely studied and associated with functional changes in P-gp expression or activity (Tanabe et al. 2001). Interindividual variation in *ABCB1* may consequently modify P-gp expression and the pharmacokinetics of corticosteroids and metabolites, contributing to the cortisol sensitivity of certain individuals and increasing the risk of developing ON. A prognostic relationship between the *ABCB1* C3435T and G2677T/A polymorphisms and cortisol-associated ON development has recently been established (Tanabe et al. 2001, Asano et al. 2003a).

The cytochrome P450 family is a group of enzymes involved in the oxidative and reductive metabolism of almost all lipid-soluble medicines. The product of the cytochrome P450 gene, *CYP3A4*, is considered to be the main cytochrome responsible for steroid metabolism (Kitada et al. 1987). *CYP3A4* also exhibits an approximate 40-fold degree of interindividual polymorphic variation, including CYP3A*1-*5 alleles, which have been associated with reduced activity of cytochrome P450 (Hsieh et al. 2001), a prognostic factor associated with increased incidence of ON and with the extent of necrotic infiltration in a rabbit model (Masada et al. 2008).

The objective of the present study was to assess the incidence of *ABCB1* C3435T and G2677T/A genotypes, and of polymorphisms of CYP3A4 (CYP3A4*4, CYP3A4*5, CYP3A4*6) in a multicenter, randomized post-corticosteroid therapy patient cohort with or without corticosteroid-associated ON. Clarification of the prevalence of specific genetic polymorphisms in corticosteroid-associated ON patients will encourage greater acknowledgement of the prophylactic measures potentially available through individualized corticosteroid administration.

## Patients and methods

### Study subjects

The study protocol was approved by the local institutional and administrative review board of the First Affiliated Hospital of Guangzhou TCM University, and all patients provided written informed consent before participation. All were from the Guangdong Province of China. 31 consecutive randomized patients who were treated with corticosteroid therapy and had documented ON, and 17 consecutive corticosteroid-therapy patients without ON were included in this study. The patients were diagnosed with ON in accordance with the criteria prescribed by Sugano et al. (2002). Patient history of corticosteroid use, exposure to hyperbaric conditions, known systemic and other concomitant diseases, and hemoglobinopathies were obtained by review of patient charts. Corticosteroid-associated ON patients were defined as those who took continuous corticosteroid medication for at least 2 months before physical and radiological examination of the hips. Patients with ON prior to corticosteroid administration or those with a history of ethanol abuse were excluded.

### Sequence analysis of the CYP3A4*4, CYP3A4*5, and CYP3A4*6 polymorphisms and the ABCB1 C3435T and G2677T/A polymorphisms

CYP3A4*4, CYP3A4*5, and CYP3A4*6 polymorphisms and *ABCB1* C3435T and G2677T/A polymorphisms were determined by sequencing polymerase chain reaction (PCR) products of the polymorphic regions from each patient, as previously described (Asano et al. 2003a). Briefly, genomic DNA for PCR was extracted from peripheral blood samples using the DNeasy Tissue Kit (Qiagen Gmbh, Hilden, Germany) following the manufacturer's instructions. Oligonucleotide primers for PCR amplification were derived from known sequences (GenBank accession number M29445 for C3435T and M29440 for G2677T/A). The sequences for the CYP primers were: forward 5'-CAC ATT TTC TAC AAC CAT GGA GAC C-3' and reverse 5'-TTT TAT ACC TGT CCC CAC CAG ATT C-3' for CYP3A4*4; forward 5'-TGT TGC ATG CAT AGA GGA AGG ATG G-3' and reverse 5'-GAT GAC AGG GTT TGT GAC AGG GG-3' for CYP3A4*5; and forward 5'-GAG CCA TAT TCT CAG AAG GGA GAT CAA G-3' and reverse 5'-CAA ACA TGT GTC GTT CTG CTA TGT GG-3' for CYP3A4*6. The sequences for the *ABCB1* primers were: forward 5'-TTC AGC TGC TTG ATG GCA AA-3' and reverse 5'-AGG CAG TGA CTC GAT GAA GG-3' for C3435T; and forward 5'-CAG GCT TGC TGT AAT TAC CC-3' and reverse 5'-TAG TTT GAC TCA CCT TCC CA-3' for G2677T/A. The PCR amplification program consisted of an initial denaturation step at 94°C for 5 min, followed by 35 cycles of denaturation at 94°C for 1 min, annealing at 58°C for 1 min and extension at 72°C for 1 min, and a final extension at 72°C for 5 min. The resulting PCR fragments were then purified and sequenced using BigDye Terminator sequencing reactions in an automated DNA sequencer (ABI 3730; Applied Biosystems).

### Statistics

Post-randomization clinical data and patient characteristics were stratified in terms of post-cortisol-associated ON patients and post-cortisol patients without ON. Statistical analysis of polymorphisms in the *ABCB1* gene was conducted using the chi-square test or Fisher's exact test. Haplotype frequencies were estimated using pairwise linkage disequilibrium coefficients calculated from estimated haplotype frequencies of C3545T and G2677T/A as previously described (Asano et al. 2003a).

## Results

### Patient characteristics

The distribution of sex and age between the ON group (31 patients) and the control group (17 patients) was similar, with 14 females and 17 males with a median age of 32 (12–58) years in the ON group, and 9 females and 8 males with a median age of 30 (19–59) years in the control group. Patients were admitted for a variety of conditions requiring corticosteroid therapy. In the ON group, the patients were admitted for trauma (4), psoriasis (2), rubeola (2), renal disease (5), mixed connective tissue disease (3), systemic lupus erythematosus (7), atopy (2), rheumatoid arthritis (1), renal transplantation (1), asthma (1), mycotic opthalmia (1), aplastic anaemia (1), or chronic pharyngitis (1). In the control group, patients were admitted for renal disease (10) or systemic lupus erythematosus (7).

### Incidence of ABCB1 C3435T and G2677T/A polymorphisms

Of the 31 ON patients who were genotyped for the *ABCB1* C3435T polymorphism, 24 had the wild-type CC genotype, 7 had the heterozygous CT phenotype, and none had the homozygous TT phenotype. Of the 17 control patients, 14 had CC, 3 had CT, and none had TT.

Of the 31 ON patients who were genotyped for the *ABCB1* G2677T/A polymorphism, 18 had the wild-type GG genotype, 9 had the heterozygous GT phenotype, 4 had the heterozygous GA phenotype, and none had any of the remaining possible polymorphic phenotypes. Of the 17 control patients, all 17 had the wild-type GG phenotype.

In the ON group, 20/31 patients carried one copy of a polymorphic allele of either C3435T (n = 7) or G2677T/A (n = 13), and 13 of all 31 patients carrying *ABCB1* polymorphic alleles developed corticosteroid-associated ON. 24 patients homozygous for the wild-type 3435CC sequence and 18 patients homozygous for the 2677GG polymorphism developed ON subsequent to corticosteroid administration.

There was significant difference between the ON and control group for SNPs in the ABCB1 G2677T/A polymorphism (p = 0.002, Table 1). Analysis of haplotypic frequencies by Fisher's exact test indicated significant linkage disequilibrium between C3435T and G2677T/A (D' = 0.03), which is in agreement with the results of Asano et al. (2003a) (Table 2).

**Table 1.** Single nucleotide polymorphisms (SNPs) of ABCB1 exon 26 (C3435T) and exon 21 (G2677T/A) (n = 48)

SNPs	SNP ON	C3435T Control	p-value **^a^**	SNP ON	G2677T/A Control	p-value **^a^**
No	24	14		18	17	
Yes	7	3	1.0	13	0	0.002

**^a^** Fisher's exact test.

**Table 2.** Chi-squared test of independence for single nucleotide polymorphisms (SNPs) of ABCB1: C3435T and G2677T/A (n = 48)

	G2677T/A SNPs			
C3435T SNPs	No	Yes	p-value **^a^**	D' **^b^**
No	15	9	0.4	0.03
Yes	3	4		
Total	18	13		

**^a^** Fisher's exact test.

**^b^** Linkage disequilibrium coefficient (D')

### Incidence of CYP3A4*4, CYP3A4*5, and CYP3A4*6 polymorphisms

Using sequence analysis, no CYP3A4*4, CYP3A4*5, or CYP3A4*6 polymorphisms were detected. These findings were discordant in part with the previously published results of Asano et al. (2003b).

## Discussion

Our results indicate a statistically significant difference between the ON and control groups regarding the ABCB1 C3435T and G2677T/A SNPs, suggesting a positive association between these genetic polymorphisms and the susceptibility of corticosteroid-associated ON. It is interesting that this result conflicts with the observation of Asano et al. (2003a), in which the C3435T (3435TT genotype, but not 3435CT genotype) and G2677T/A (2677TT, 2677TA, and 2677AA genotypes) SNPs were associated with a reduced risk of corticosteroid-associated ON. Ethnic background should probably be considered to be the primary cause of this diversity. To date, 28 SNPs in the *ABCB1* gene have been reported at 27 positions in Caucasians and Africans (Hoffmeyer et al. 2000, Kim et al. 2001, Siegmund et al. 2002), of which only a few C3435T SNPs were associated with P-gp expression, function, or increased plasma concentration. Other studies have also found a diversified allelic frequency in the wobble SNP in C3435T, with Caucasians and Japanese showing frequencies different from those of Africans (Saeki et al. 1993). The involvement of the 3435T variant allele has been reported previously by Siegmund et al. (2002), in which healthy Japanese patients harboring the allele had lower serum concentrations of digoxin after a single injection as compared to wild-type subjects. Other studies have shown conflicting results, with a lack of genotype-phenotype correlation between C3435T SNPs and cyclosporine efficacy in renal transplantation patients, indicating that C3435T is not the only polymorphism involved in P-gp expression (Sakaeda et al. 2001). However, recent studies have shown that carriers of the *ABCB1* 3435T allele have enhanced oral clearance of cyclosporine compared to Caucasian individuals with the 3435CC genotype (von Ahsen et al. 2001), and they have reduced expression of intestinal CYP3A4 mRNA (Yates et al. 2003).

A statistically significant linkage disequilibrium between the C3435T and G2677T/A SNPs was identified in both this study and that of Asano et al. (2003a), corroborating the involvement of the *ABCB1* C3435T and G2677T/A SNPs in the pathogenesis of corticosteroid-associated ON. However, whether the involvement is a risk factor for or against corticosteroid-associated ON remains to be determined. Although the physiological role of P-gp remains elusive, interindividual expression of the highly polymorphic ABCB1 gene is likely to contribute to variability in the pharmacokinetics and pharmacodynamics of many drugs (Saeki et al. 1993) in addition to increased susceptibility to cancer (Siegsmund et al. 2002). C3435T and G2677T/A SNPs do not involve amino acid substitution and do not functionally influence P-gp expression directly (Siegmund et al. 2002). Thus, their functional significance may be that they are involved in post-transcriptional mRNA processing and affect the quality or quantity of P-gp protein expression.

The frequencies of the C3435T alleles in our test groups were much different from those reported for normal healthy Han Chinese populations (reviewed in Li et al. 2006). Previous studies of Han Chinese populations from Singapore, southwest China, and Northern China showed a typical Hardy-Weinberg distribution of genotypes. In contrast, our data show a low frequency of T alleles. It is likely that our results may have been affected by both sampling error due to low sample size and by the fact that the samples came from unhealthy individuals. Similar conclusions may be drawn regarding the G2677T/A allele, based on its strong linkage to the C3435T allele.

The *CYP3A* subfamily of cytochrome P450 proteins are found most abundantly in the liver and intestine, accounting for approximately 95% of the *CYP3A* mRNA-pool in Caucasians (Koch et al. 2002). The protein products of *CYP3A4* and *CYP3A5* are preferentially involved in the catalysis of exogenous and endogenous compounds. Contrary to the expected SNP variability of *CYP3A* genes (Hsieh et al. 2001), all of the subjects in our patient cohort were homozygous wild-types. Certain alleles of these genes (CYP3A4*4 and CYP3A4*5) are associated with clinically relevant changes in drug clearance (Felix et al. 1998, Thervet et al. 2003). Our study design did not allow for measurement of the systemic clearance of *CYP3A* substrate drugs in relation to development of ON; however, recent studies have shown that the CYP alleles we examined are rare in the Chinese population (Hsieh et al. 2001, Wen et al. 2004). Instead, the Chinese population contains a number of novel CYP mutations that are rare in other populations (Du et al. 2006). We suggest evaluation of the roles of other *CYP3A* genes in relation to ON and control groups, with clearly identifying the substrate regiments of the gene products in the future.

Interestingly, all the patients who did not develop ON had either kidney disease or lupus. Both kidney disease (Naud et al. 2008) and lupus (Tsujimura et al. 2005) can increase P-gp activity. It is possible that these diseases counteracted any genetic influences on corticosteroid clearance by upregulating P-gp expression, thus providing protection against ON.
